# Identification of Gingival Microcirculation Using Laser Doppler Flowmetry in Patients with Orthodontic Treatment—A Longitudinal Pilot Study

**DOI:** 10.3390/medicina57101081

**Published:** 2021-10-10

**Authors:** Martha Alicia Laredo-Naranjo, Nuria Patiño-Marín, Gabriel Alejandro Martínez-Castañón, Carlo Eduardo Medina-Solís, Carolina Velázquez-Hernández, Nereyda Niño-Martínez, Marco Felipe Salas Orozco

**Affiliations:** 1 Clinical Research Laboratory, Program of Doctorate in Dental Sciences, Faculty of Stomatology, Autonomous University of San Luis Potosí, San Luis Potosí C.P. 78000, Mexico; aliciaa.laredo@uaslp.mx (M.A.L.-N.); mtzcastanon@fciencias.uaslp.mx (G.A.M.-C.); a33302248@alumnos.uaslp.mx (C.V.-H.); marcco-salas@hotmail.com (M.F.S.O.); 2 Academic Area of Dentistry, Health Sciences Institute, Autonomous University of Hidalgo State, Pachuca C.P 42000, Mexico; cemedinas@yahoo.com; 3 Materials Characterization Laboratory, Program of Doctorate in Dental Sciences, Faculty of Science, Autonomous University of San Luis Potosí, San Luis Potosí C.P. 78000, Mexico; nereyda.nino@uaslp.mx

**Keywords:** gingival microcirculation, laser-doppler flowmetry, orthodontic treatment

## Abstract

*Background and Objectives*: Orthodontic tooth movement is associated with inflammatory responses. The aim of this study was to identify gingival microcirculation using laser Doppler flowmetry in patients with orthodontic treatment. *Materials and Methods*: A longitudinal pilot study was performed. The participants were selected using a non-probability consecutive sampling. Of the twenty-five subjects, a total of six (four women and two men) complied with the criteria. Before and during the treatment, the oral hygiene index, gingival index, probing depth, level of epithelial attachment, and gingival microcirculation were evaluated with laser Doppler flowmetry (integrated parameters: 1. integrated primary basal flow (IPBF), 2. integrated total secondary real flow (ITSRF), and 3. difference between integration (DBI)) in all of the participants). *Results*: (a) An increase in gingival blood flow was identified at all time intervals with different arches during orthodontic treatment. (b) The IPBF and ITSRF (with treatment) identified after 20 min (treatment initial stage) were compared with the different time intervals, and we observed an increase in gingival perfusion at the 24th, 48th, and 72nd hours in some arches. (c) In the DBI, we found statistically significant differences (*p* < 0.005) in the Nitinol group of 0.016 inches among all the time intervals (24 h, 48 h, and 72 h) within the 30-day interval, observing a flow increase three times greater than the basal flow after 30 days. *Conclusions*: Healthcare professionals must identify the inflammatory processes in treatment to observe and discontinue use of harmful methods in clinical practice.

## 1. Introduction

Several researchers have published studies about the presence of poor oral hygiene, gingivitis, and an increase in probing depth and attachment level loss after orthodontic treatment [1,2,3].

The use of orthodontic appliances, particularly fixed appliances, can favor an increase in periodontal tissue inflammation; therefore, patients receiving orthodontic treatment should be kept under strict control and undergo regular hygiene sessions [1,2,3].

Pain, discomfort, dental caries, periodontal diseases, root resorption, time duration, and appliances are variables that the clinician should identify during the treatment [4,5].

Orthodontic tooth movement is associated with inflammatory responses, such as (a) vasodilation with increased vessel permeability and blood flow; (b) exudation of fluids; and (c) leukocyte migration into extravascular spaces [4,5].

The degeneration of endothelial cells and changes in blood flow, vascular caliber and vascular permeability in periodontal ligaments, and alveolar bones and gingiva may be the first signs of the onset of pathological events with the application of orthodontic forces to the teeth during specific periods of time [1,4,6].

Thus, health professionals must identify the optimal force, considering the magnitude and temporal characteristics (continuous vs. intermitted; constant vs. declining) without tissue damage and with the maximum patient comfort during the provision of treatment.

A better understanding of the relationship between gingival microcirculation and orthodontic treatment will expand our knowledge and might strengthen the clinical practice.

Laser Doppler flowmetry is a sensitive, continuous, non-invasive, frequency-responsive method that can be used in real time for perfusion measurements in undisturbed microcirculation [7,8,9].

Therefore, the aim of this study was to identify gingival microcirculation using laser Doppler flowmetry in patients with orthodontic treatment (longitudinal pilot study).

## 2. Materials and Methods

A longitudinal pilot study was performed from June 2017 to January 2020 in San Luis Potosi, Mexico. Based on the ethical principles of the Declaration of Helsinki, informed and voluntary written consent was obtained from patients’ parents or from the patients themselves prior to the beginning of the study. The study was approved by the Research Ethics Committee of the Autonomous University of San Luis Potosi. 

### 2.1. Study Population and Selection of the Sample

**Figure 1:**
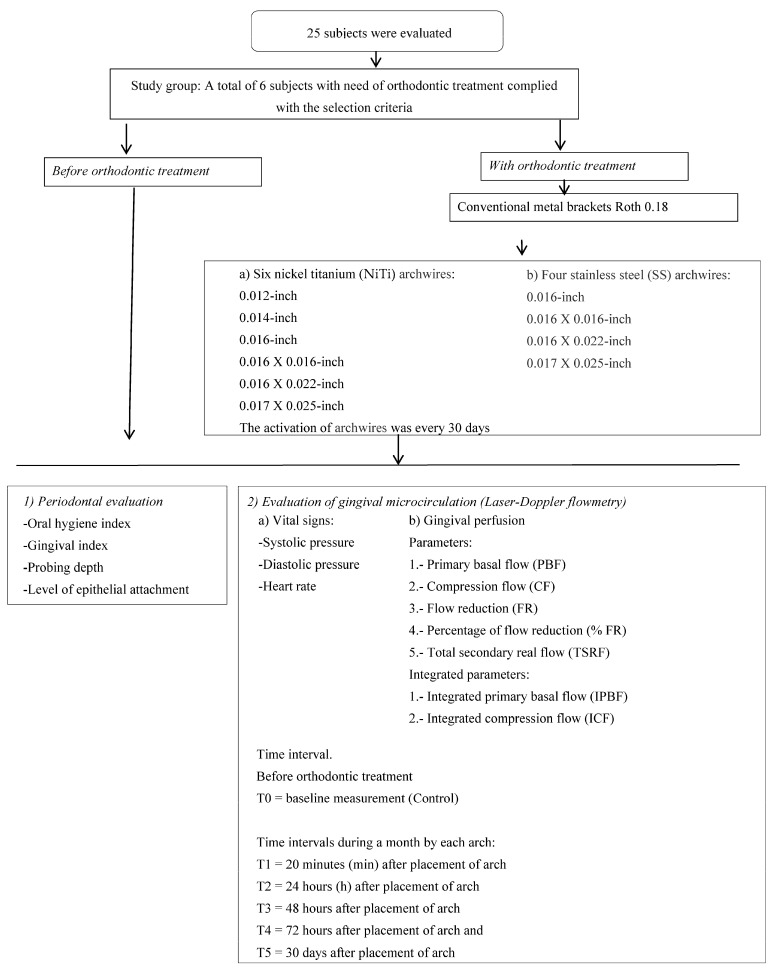
Protocol of the evaluation for periodontal tissues and gingival microcirculation using laser Doppler flowmetry in patients before and during orthodontic treatment.

The participants were selected using non-probability consecutive sampling. Twenty-five subjects requiring orthodontic treatment were evaluated to identify a diagnosis and a treatment plan (Figure 1).

For the diagnosis and treatment plan of each subject, the following were obtained: clinical history; clinical examination (extraoral and intraoral); and records, such as: (1) study models, (2) intraoral and extraoral photographs, (3) radiographs (panoramic, periapical, and lateral radiographs of the skull); and (4) computed tomography (cone beam).

Of the 25 subjects, a total of six (four women and two men) complied with the following inclusion criteria: (1) subjects without diagnosed systemic diseases; (2) both genders; (3) 15–28 years old; (4) subjects not using drugs; (5) non-smokers; and (6) subjects requiring orthodontic treatment. Then, the following oral cavity criteria were set: (1) patients with complete permanent dentitions, (2) Class I malocclusion (Angle’s classification of malocclusion); (3) dental crowding in both arches (mean = 4 to 5 mm); (4) subjects without the need for extraction of premolars; (5) mesofacial biotype; (6) skeletal Class I malocclusion; (7) presenting with an A point–nasion–B point (ANB) angle between 2 and 5 degrees; (8) presenting with an interincisal angle between 120 and 146 degrees; (9) healthy periodontal tissue; (10) good oral hygiene; and (11) intact teeth. In addition, the following exclusion criteria were set: (1) patients with previous orthodontic or periodontal treatment; (2) pregnancy; and (3) evident genetic diseases. The elimination criteria were as follows: (1) a technical impossibility to reach a perfusion value of about 1/5 of its control value in two consecutive recordings during gingival compression and (2) a technical inability to evaluate the variables.

### 2.2. Calculation of Sample Size

The calculations to identify the sample size were obtained from a previous study [10]. We used the formula of studies with dependent measures in a group to calculate the size. The sample size that we identified with a power of 0.90 and a significance level of 0.05 was a minimum of four subjects [11].

### 2.3. Study Group

The subjects requiring orthodontic treatment and who complied with the selection criteria constituted the study group. 

#### Orthodontic Treatment (Study Group)

All of the patients were treated with conventional Roth 0.18 metal brackets (Mini Master Series, AO, American Orthodontics, Sheboygan, Wisconsin, USA) to place six preformed nickel–titanium arches (0.012, 0.014, 0.016, 0.016 × 0.016, 0.016 × 0.022, and 0.017 × 0.025) (Niti Memory Wire Form1 Force 1, AO, American Orthodontics)) and four stainless steel arches (0.016, 0.016 × 0.016, 0.016 × 0.022, and 0.017 × 0.025) (Stainless Steel Wire NA Form1, AO, American Orthodontics). Each arch wire was held in each bracket slot with an elastomeric module (AO, American Orthodontics). Orthodontic activation occurred every 30 days, and the duration of the treatment was two years (Figure 1).

### 2.4. Protocol of Evaluation for Periodontal Tissues and Gingival Microcirculation Using Laser Doppler Flowmetry

#### Periodontal Evaluation

Before and during the treatment, periodontal tissues were evaluated in all of the participants each month with the following indices: (a) Oral hygiene was assessed using the plaque and calculation index of Greene and Vermillion, and the average was calculated and interpreted as good (0–1.2), regular (1.3–3.0), and bad (>3.1); [12]. (b) The presence or absence of gingivitis was evaluated by the gingival index in 3 areas (gingival papillae, gingival margin, and attached gingiva). Each area was scored as 1 or 0 based on the presence or absence of inflammation, respectively. With the results of this index, we were able to calculate a mean [13]. (c) The probing depth and level of epithelial attachment were recorded using a calibrated periodontal probe in millimeter scale (Hu-Friedy, Chicago, IL, USA). The probing depth was evaluated from the gingival margin to the base of the gingival pocket, considering a healthy sulcus as <3 mm. The attachment level was evaluated from the cement–enamel junction to the base of the sulcus, considering a healthy sulcus as <2 mm. The evaluation was carried out in all of the permanent patients’ teeth. The presence or absence of periodontal disease was determined based on all of the periodontal indices (Figure 1) [14].

### 2.5. Gingival Microcirculation Using Laser Doppler flowmetry

#### 2.5.1. Fabrication of the Probe Stabilizer with the Laser Doppler Technique

To perform a measurement with the PeriFlux 5000 (Manufactured by Perimed AB, Datavägen, Järfälla, Sweden) unit 5010 laser doppler blood perfusion monitoring (LDPM) system (laser Doppler technique), we used the stabilizer (holder) of the probe (Probe 457, Manufactured by Perimed AB, Datavägen, Järfälla, Sweden) with a rigid acetate (0.20 gauge), diameter of 10 mm, and height of 8 mm (Splint Sheets, Ultradent, South Jordan, UT, USA). The stabilizer was made based on the study models of each subject with a heat/vacuum tray-forming machine (Sta Vac, Buffalo Dental, Syosset, NY, USA). In the acetate, we integrated a mini holder to place the laser Doppler probe and perform the measurement [15].

#### 2.5.2. Subject Position

1. Each subject was placed in a comfortable decubitus supine position on a dental chair. 2. The head was placed with a slight neck hyperextension. 3. The subject was given a full explanation of the procedure and was asked to relax during it. 4. After 20 min of patient stabilization, a soft tissue retractor (Morita Co., Osaka, Japan), the probe stabilizer of the laser, and the laser Doppler probe (Probe 407-1, Perimed AB) were placed in the oral cavity to obtain gingival perfusion records [15].

The vital signs, sites of perfusion and phases, parameters, and time intervals were evaluated with the following protocol:

1. Evaluation of vital signs

The systolic pressure, diastolic pressure, and heart rate were identified in patients during the gingival perfusion measurement with a M8000 monitor (Multiparameter patient monitor M8000, Biolight CO. LTD., coastal Zhuhai, China) (Figure 1) [15,16].

2. Sites of perfusion and phases of evaluation

With the calibrated equipment, two continuous perfusion measurements were carried out with a 5-min interval between each measurement. Four sites of perfusion per patient were evaluated, one site on the anterior teeth (vestibular papilla) and one site on the posterior teeth (vestibular papilla) on the maxilla and mandible with a total of four sites. In each measurement, three phases were identified: the control phase (basal flow without compression) of 40 s, the compression phase of 20 s, and the post-compression phase of 40 s. In the compression phase, the perfusion was decreased to about 1/5 of its control perfusion value (baseline measurement) with a fixed and sustained compression with a PCPUN-15 periodontal probe (Hu-Friedy, Chicago IL, USA) on the alveolar mucosa. During the post-compression phase, the compression was eliminated [15,16].

3. Parameters

We evaluated the following parameters: 1. primary basal flow (PBF) = average perfusion during the control phase; 2. compression flow (CF) = average perfusion during sustained gingival compression; 3. flow reduction (FR) = PBF—CF; 4. percentage of flow reduction (% FR); and 5. total secondary real flow (TSRF) = average perfusion during the post-compression phase. Integrated parameters: 1. integral PBF (IPBF) = area under the curve during the control phase; 2. integral CF (ICF) = area under the curve during the compression phase; 3. integral TSRF (ITSRF) = area under the curve during the post-compression phase; 4. difference between integration (DBI) = IPBF—ITSRF. Negative values represent an increase in blood flow. The data obtained in the study were registered with a frequency of 32 Hz and analyzed using PSW Perisoft 1.30 V software, which allowed us to obtain values of the area below the curve. The flows are expressed in relative perfusion units (PUs) (Figure 1) [15,16].

4. Time intervals

Before orthodontic treatment, a baseline measurement (T0 = control) was performed. With orthodontic treatment, the following measurements were obtained each month per arch: T1 = 20 min after placement of arch, T2 = 24 h after placement of arch, T3 = 48 h after placement, T4 = 72 h after placement, and T5 = 30 days after placement of arch (Figure 1).

5. PeriFlux System

The gingival perfusion recordings were obtained using the Perimed AB (PeriFlux System, Datavägen, Järfälla, Sweden) consisting of a PF 5001 main unit, a PF 5010 LDPM unit (Class 1 laser, 780 nm, near-infrared laser diode, power = 1 mW, time constant = 0.2 s, bandwidth = 20 Hz–20 kHz, validated electronic linearizer (Nilsson 1984)), a Probe 457 (10 mm diameter, 0.25mm fiber separation), and a PF1001 calibration device (PF1001 Refill Motility Standard, Perimed, Datavägen, Järfälla, Sweden) [15,16].

### 2.6. Statistical Analysis

The examiner was calibrated to measure the variables. (a) The intraclass correlation coefficients and kappas obtained during the calibration were greater than 0.80 [17]. (b) The categorical variables were reported with frequencies and percentages, and the continuous variables were reported with means, standard deviations, and rank (man ± SD (rank)). (c) The Shapiro–Wilk and Brown–Forsythe tests were performed to determine the distribution of the variables. (d) To determine the differences among variables in the study group, we used the Wilcoxon signed-rank test (two dependent samples). As a result of the analysis, 6,000,000 gingival perfusion data items were generated per patient, with a total of 36,000,000 perfusion data items considering all of the study patients (*n* = 6). JMP (SAS Institute, Cary, NC, USA) version 9 and Stat View (SAS Institute, Cary, North Carolina, USA) were used for the statistical analysis, and the statistical significance was set at *p* < 0.05.

## 3. Results

Twenty-five subjects were evaluated in the present study. Four women and two men (age: 22 ± 5.4 (15–28)) complied with the selection criteria. We identified the following variables in every participant’s oral cavity: (a) patients with Class I molar and canine malocclusion; (b) dental crowding in the upper arch of 5 ± 3.2 mm; (c) crowding in the lower arch of 4 ± 1.5mm; (d) mesofacial biotype; (e) skeletal Class I malocclusion; (f) ANB angle of 3 ± 1 (2–5); (g) interincisal angle of 129 ± 8.0 (122–146); (h) 50% with a straight facial profile and 50% with a slightly convex facial profile; and (i) subjects without the need of extractions of premolars. The periodontal tissues and gingival microcirculation were evaluated in all of the participants. 

### 3.1. Periodontal Evaluation

Before starting the treatment, we identified the oral hygiene index = 0 ± 0.07 (0.0–1, good hygiene.); gingival index = 0.09 ± 0.2 (0–2) absence; probing depth = 1 ± 0.0 (1–1); and level of epithelial attachment = 1 ± 0.0 (1–1) healthy sulcus. During the orthodontic treatment, we observed an oral hygiene index of 0.06 ± 0.06; a gingival index of 0.3 ± 0.1; a probing depth of 1 ± 0.1; and a 1 ± 0.1 level of epithelial attachment. The absence of periodontal disease in the study subjects was identified via these indices.

### 3.2. Evaluation of Gingival Microcirculation Using Laser Doppler Flowmetry. Vital Signs and Gingival Microcirculation Were Identified in the Patients

(a) Vital Signs. Systolic pressure = 125 ± 14 (78–178); diastolic pressure = 80 ± 9.6 (53–112); and heart rate = 95 ± 14 (54–132). We observed normal ranges in patients during the study.
(b) Gingival microcirculation. We evaluated the patients’ microcirculation before and during treatment.

#### 3.2.1. Gingival Microcirculation before Treatment

The following parameters were observed in the study group: Basic parameters: 1. primary basal flow (PBF) = 164 up ± 60; 2. compression flow (CF) = 40 up ± 16; 3. flow reduction (FR) = 123 up ± 4; 4. percentage of flow reduction (% FR) = 74 ± 4; and 5. total secondary real flow (ITSRF) = 157 up ± 97. Integrated parameters: 1. integrated primary basal flow (IPBF) = 6664 up ± 2927; 2. integrated compression flow (ICF) = 1500 up ± 800; 3. integrated total secondary real flow (ITSRF) = 7188 up ± 3394; and 4. difference between integration (DBI) = −524 up ± 3160.

#### 3.2.2. Gingival Microcirculation with Treatment

Considering all of the arches for the different time intervals of the study group, the perfusion parameter averages were as follows: Basic parameters: 1. primary basal flow (PBF) = 186 up ± 73; 2. compression flow (CF) = 60 up ± 28; 3. flow reduction (FR) = 132 up ± 5.7; 4. percentage of flow reduction (% FR) = 70 ± 8.7; 5. total secondary real flow (ITSRF) = 207 up ± 83. Integrated parameters: 1. integrated primary basal flow (IPBF) = 7688 up ± 3713, 2. integrated total secondary real flow (ITSRF) = 8505 up ± 4323, and 3. difference between integrated (DBI) = −665 up ± 1583.

Table 1 shows the evaluation of integrated primary basal flow (IPBF) in patients with orthodontic treatment, including Nitinol and steel arches at different time intervals. When comparing the IPBF with and without treatment at the different time intervals for the Nitinol and stainless steel arches, we found statistically significant differences (*p* = 0.0006) between the IPBF without treatment (6664 up) and the IPBF of stainless steel arches (0.017 × 0.025 inches at 48 h), identifying higher values in the stainless steel group (12,372 up).

The IPBF in patients with treatment identified after 20 min was compared with the arch time intervals, observing statistically significant differences (*p* < 0.005) for the time durations of 24 h (Nitinol 0.017 × 0.025 inches = 10,807; stainless steel: 0.016 × 0.016 inches = 9901 up and 0.017 × 0.025 inches = 10,624 up), 48 h (Nitinol 0.016 × 0.016 inches = 10,878 up; stainless steel 0.017 × 0.025 inches = 12,372 up), and 72 h (Nitinol 0.016 × 0.022 inches = 10,198 up; stainless steel 0.017 × 0.025 inches = 8565 up), identifying an increase in gingival perfusion starting at the 24th hour and continuing up to the 72nd hour in some arches. When comparing the Nitinol arches with the stainless steel ones and when comparing round arches with square–rectangular ones, we did not find statistically significant differences in the five time intervals of the various arches.

The integrated total secondary real flow (ITSRF) in patients with orthodontic treatment for the different time intervals can be seen in Table 2. When comparing the ITSRF in patients with and without treatment, we found statistically significant differences (*p* < 0005) for time intervals of (a) 24 h (Nitinol 0.017 × 0.025 inches = 12,041 up; stainless steel 0.017 × 0.025 inches = 11,641 up), (b) 48 h (Nitinol 0.016 × 0.016 inches = 11,752 up; stainless steel: 0.016 × 0.022 inches = 12,616 up and 0.017 × 0.025 inches = 12,821 up), and (c) 72 h (Nitinol 0.016 × 0.022 inches = 11,244 up), observing an increase in gingival perfusion at the 24th, 48th, and 72nd hours. On the other hand, when comparing the ITSRF after 20 min with the different time intervals of all of the arches, we found statistically significant differences (*p* < 0.005) in the same time intervals and arches identified with IPBF. We also found differences in (1) Nitinol 0.016 × 0.022 inches = 10,196 up (24 h) and 9244 up (48 h) and (2) stainless steel 0.016 × 0.022 inches = 12,616 up (48 h), observing an increase in gingival perfusion from the 24th hour to the 72nd hour. Moreover, for variable IPBF, when comparing the Nitinol arches with the stainless steel ones and when comparing the round arches with the square–rectangular ones, we did not find statistically significant differences.

Table 3 shows the difference between integration (DBI) in patients with orthodontic treatment (Nitinol and stainless steel arches) at different time intervals. When comparing the DBI with and without treatment and when comparing the DBI after 20 min with the different time intervals of all of the arches, we found statistically significant differences (*p* < 0.005) for the 0.016-inch Nitinol group after 30 days (−2,178 up), observing high perfusion values in the 30-day interval.

A comparison between time intervals in hours and days for the DBI in patients with orthodontic treatment can be seen in Table 4. We found statistically significant differences between the time interval of the 24th (−647 up), 48th (−852 up), and 72nd (−643 up) hours within the 30-day interval (−2178 up) for the Nitinol arch group of 0.016 inches, observing a perfusion increase after 30 days. In addition, for the variables IPBF and ITSRF, when comparing the DBI between the Nitinol and stainless steel arches and the round arches with the square–rectangular ones, we did not find statistically significant differences in the five time intervals.

## 4. Discussion

The aim of the present study was to identify gingival microcirculation using laser Doppler flowmetry in patients with orthodontic treatment (a longitudinal pilot study). The analysis of gingival microcirculation, laser Doppler flowmetry, and orthodontic treatment could provide information to facilitate decision making in clinical practice. 

### 4.1. Gingival Microcirculation 

Gingival microcirculation involves a combination of (a) physical processes, such as the transport of blood flow, blood vessel mechanics, and diffusive mass transport, and (b) biological processes, such as active cellular responses to physical and biochemical signals [18,19].

At the cellular level, vascular smooth muscle cells interact with the endothelium, which releases mediators involved in relaxation and contraction [19]. During pathological events such as gingival inflammation, the first microvascular changes are dilatation and increased blood flow. Gingivitis can end the restoration of normal tissue morphology and function, and with the evolution of chronic inflammation, it can cause tissue destruction. Investigators have employed a variety of techniques to examine gingival microvascular function. Laser Doppler flowmetry (LDF) is a method that identifies the gingival microvascular dynamics [16,20].

### 4.2. Laser Doppler Flowmetry (LDF) 

When an infrared laser is exposed to red blood cells in microvessels, reflection and scattering occur, and a Doppler shift causes a frequency change between the incidence and reflection in proportion to the blood flow [15,16,21].

LDF is a sensitive, continuous, non-invasive, frequency-responsive method that can be used in real time for perfusion measurements in undisturbed microcirculation. However, some drawbacks that have been reported by different researchers who have used this methodology are as follows: (a) the provision of average blood cell flux (cells/m2/s) linear readout in relative perfusion units (PU), (b) between-subject variability, and (c) uncertainty regarding whether a given readout value of relative PU represents much or little perfusion in a subject. A small sample volume, the optical properties of the sample volume, and variations in microvascular hematocrit are some possible explanations for the drawbacks of this technique. Regardless of the reason, the point that must be emphasized is that, to date, there is no average calibration factor able to convert relative PU to absolute flow value [7,8,9,15,16,21]. On the other hand, with the LDF technique, we can decrease the variability between subjects using reactivity tests.

#### Reactivity Tests

Mechanical stimuli (occlusion of an artery and pressure applied to the skin), thermal provocation (heating and cooling), electrical stimuli, and local administration of pharmacological agents have been used as reactivity tests [18,19,21].

Post-occlusive reactive hyperemia is a mechanical stimulus. Arterial occlusion has been proposed as a test of endothelial microvascular function. In human tissues, a transient increase in flux is observed immediately after the release of an occlusion (vasodilator response). Sensory nerves and metabolites are the major contributors in the responses of hyperemia. Post-occlusive reactive hyperemia allows one to obtain a baseline measure (measured before the occlusion) and an experimental measure (release of an occlusion), and with both basal and experimental measurements, each subject is also their own control [15,16,18,19,21,22,23,24,25].

Arterial occlusion (pressure applied) can be controlled, reproduced, and quantified. To achieve control and reproduction of the arterial occlusion (pressure applied to periodontal tissue) in all of the study participants, the strategy used was to reduce one-fifth of the IPBF in every patient with tissue pressure for 20 s. With this technique, we could obtain perfusion measures with intraobserver reproducibility without within-subject differences [15,16].

### 4.3. Gingival Microcirculation Using Laser Doppler Flowmetry in Patients with Orthodontic Treatment (Longitudinal Pilot Study) 

Several authors have published studies regarding some changes in the degeneration of endothelial cells and vascular networks in periodontal tissues as a result of the application of orthodontic forces to the teeth during specific periods of time [1,4]. Some of these changes are as follows: (1) release of neuropeptides (nociceptive and vasoactive) from paradental afferent nerve endings; (2) interaction of vasoactive neuropeptides with endothelial cells; (3) adhesion of leukocytes to activated endothelial cells; (4) plasma extravasation from dilated blood vessels; (5) migration of leukocytes into extravascular space; (6) synthesis and release of molecules (cytokines and growth factors) by leukocytes; and (7) interaction of various types of cells with molecules released by leukocytes [4,5,6,26,27].

In relation to tooth movement and periods of time with mechanical forces, some scholars have suggested that the initial tooth movement lasts from 24 h to 2 days. During the second phase, tooth movement stops for 20 to 30 days (removal of necrotic tissue), and after 40 days, the movement continues. Cellular and tissue reactions start during the initial phase of tooth movement, immediately after force application. Because of the compression of fibers and cells in periodontal ligament pressure and tension areas, the recruitment of osteoclasts and osteoblasts, extravasation, and chemoattraction of inflammatory cells begin. Only the removal of the necrotic tissue and bone resorption from adjacent marrow spaces allow the resumption of tooth movement. In areas of periodontal ligament tension, quiescent osteoblasts (bone surface lining cells) are enlarged and start producing a new bone matrix. Simultaneously, periodontal ligament fibroblasts in tension zones begin multiplying and remodeling their surrounding matrix. Four days after the initial force application, the pressure sides of teeth present collagen fibers without proper orientation. Here, irregular bone surfaces are found, which means a direct or frontal resorption. The removal of the necrotic tissue is a continuous process that occurs during tooth displacement [26,27,28].

The activation of cells that participate in the modeling and remodeling of the tissue is associated with vascular changes, and for this study we evaluated the following flow parameters: (1) integrated primary basal flow (IPBF; basal measure of post-occlusive reactive hyperemia); (2) integrated total secondary real flow (ITSRF; post-occlusive reactive hyperemia); and (3) difference between integration (DBI; difference between IPBF and ITSRF).

(a) When comparing the IPBF with and without treatment, we found statistically significant differences (*p* = 0.0006) in the 0.017 × 0.025 inch stainless steel arches after 48 h. In relation to ITSRF, we found statistically significant differences (*p* < 0005) for 24 h, 48 h, and 72 h time intervals in different arches, and for the DBI, we found statistically significant differences (*p* < 0.005) in the 0.016-inch Nitinol group between the variable with no treatment and the time interval of 30 days. In all of the comparisons, we observed an increase in gingival perfusion with treatment.
(b) The IPBF and ITSRF (with treatment) identified after 20 min (treatment initial stage) were compared with the different time intervals, and we observed an increase in gingival perfusion at the 24th, 48th and 72nd hours in some arches.
(c) In the DBI, we found statistically significant differences (*p* < 0.005) for the 0.016-inch Nitinol group among all of the time intervals (20 min, 24 h, 48 h, and 72 h) within the 30-day interval, observing a flow increase that was three times greater than the basal flow after 30 days.

Researchers have reported that Nitinol arches produce constant and continues forces to generate greater tooth movement in comparison to stainless steel arches; this could be a possible explanation for the increase in the gingival perfusion of these arches [29,30,31]. 

After researching papers from the last 25 years, we only found one article focused on the identification of gingival microcirculation (gingival blood flow) using laser Doppler flowmetry in patients with orthodontic treatment. Barta et al. published an article entitled “Changes in gingival blood flow during orthodontic treatment”. The aim of this study was to measure microcirculation in gingiva and its changes with time (over a period of six months) during orthodontic treatment using LDF. They observed that in most patients, the first values decreased (during the first month) and then increased gradually up to the sixth month. Our results coincide with those of Barta et al. because, in our study, we observed an increase in gingival perfusion at all of the time intervals with different arches [29]. The vascular morphology is related to blood flow changes (relaxation and contraction), and these changes may be the first sign of the onset of pathological events; therefore, it is helpful to provide information related to blood flow in clinical practice.

### 4.4. Longitudinal Pilot Study

We identified a scarcity of articles related to gingival blood flow (using laser Doppler flowmetry) in patients receiving orthodontic treatment. Longitudinal pilot studies could provide information on this topic, thereby facilitating further research [29,32].

### 4.5. Clinical Application

In this study, we observed an increase in gingival perfusion during orthodontic treatment. The increase in perfusion was identified in arches of Nitinol and steel at different time intervals (20 min, 24 h, 48 h, 72 h, and 30 days). 

However, only in the 0.016-inch Nitinol group did we observe a flow increase that was three times greater than the basal flow at 30 days. Therefore, health personnel should (1) observe oral health and (2) when necessary, reassess the treatment in relation to arch selection, use of arches, and treatment times. These strategies could be used to identify and discontinue the use of harmful methods in clinical practice.

## 5. Conclusions

An increase in gingival blood flow was identified at all of the time intervals with different arches during orthodontic treatment. A greater increase was observed in the Nitinol arch group of 0.016 inches in the 30-day interval.

## Figures and Tables

**Table 1:** Evaluation of the integrated primary basal flow (IPBF) in patients with orthodontic treatment (Nitinol and steel arches) at different time intervals.

Time Intervals	20 min	24 h	48 h	72 h	30 d
Arch groups (*n* = 24 sites by arch)			Mean ± SD (PU)		
Nitinol (caliber)					
0.012 inches	5296 ± 2382	6454 ± 2340	6929 ± 3515	5885 ± 3593	6563 ± 2645
0.014 inches	4712 ± 2424	7989 ± 2905	8163 ± 3759	5674 ± 1775	5930 ± 2948
0.016 inches	5570 ± 1924	5412 ± 2029	7039 ± 3045	6795 ± 3331	6745 ± 3198
0.016 × 0.016 inches	6097 ± 3089	9927 ± 4226	10,878 ± 4386 *	6912 ± 3378	7715 ± 3552
0.016 × 0.022 inches	4882 ± 3520	8469 ± 5690	8543 ± 3428	10,198 ± 3506 *	7789 ± 3409
0.017 × 0.025 inches	6713 ± 4131	10,807 ± 5485 *	8932 ± 5520	7555 ± 2489	7858 ± 3727
Mean ± SD (*n* = 144 sites)	5545 ± 2911	8176 ± 3779	8414 ± 3942	7169 ± 3012	7100 ± 3246
Stainless steel (caliber)					
0.016 inches	7568 ± 5053	6542 ± 2635	6934 ± 4749	6169 ± 3110	7046 ± 3529
0.016 × 0.016 inches	5547 ± 3947	9901 ± 4034 *	6561 ± 2750	7181 ± 3404	7395 ± 2964
0.016 × 0.022 inches	8404 ± 6333	10,884 ± 5271	10,910 ± 3706	9602 ± 4292	7088 ± 2771
0.017 × 0.025 inches	6564 ± 4878	10,642 ± 4827 *	12,372 ± 5221 ^§,^*	8565 ± 4628 *	6064 ± 2887
Mean ± SD (*n* = 96 sites)	7020 ± 5052	9492 ± 4191	9194 ± 4106	7879 ± 3858	6898 ± 3037
Total mean ± SD (*n* = 240 sites)	6282 ± 3981	8834 ± 3985	8804 ± 4024	7524 ± 3435	6999 ± 3141
Mean ± SD for round arches (*n* = 96 sites)	5786 ± 2945	6599 ± 2477	7266 ± 3767	6130 ± 2952	6571 ± 3080
Mean ± SD for square–rectangular arches (*n* = 144 sites)	6367 ± 4316	10,105 ± 4922	9699 ± 4168	8335 ± 3616	7318 ± 3218

*n* = 6 patients; SD, standard Deviation; PU, relative perfusion units; min, minutes; h, hours; d, days; *n* = 24 sites of perfusion (vestibular papilla) (1 site on anterior teeth and 1 site on posterior teeth on the maxilla and mandible with a total of 4 sites per patient). Considering 6 patients, we obtained a total of 24 sites per arch, with a total of 240 sites for the 10 treatment arches. To establish the differences among variables in a group, the Wilcoxon signed-rank test was used. § Statistically significant differences (*p* < 0.005) between the IPBF with no treatment (6664 up) and that with treatment. * Statistically significant differences (*p* < 0.005) in IPBF after 20 min compared to the different time intervals of all of the arches.

**Table 2:** Evaluation of the integrated total secondary real flow (ITSRF) in patients with orthodontic treatment (Nitinol and steel arches) at different time intervals.

Time Intervals	20 min	24 h	48 h	72 h	30 d
Arch groups (*n* = 24 sites by arch)			Mean ± SD (PU)		
Nitinol (caliber)					
0.012 inches	6544 ± 3042	7401 ± 2821	7487 ± 3911	6711 ± 3881	7130 ± 3143
0.014 inches	5488 ± 2844	9051 ± 3009	9439 ± 4484	6298 ± 2098	6688 ± 4868
0.016 inches	6072 ± 2354	6059 ± 2011	7891 ± 3305	7438 ± 3305	8923 ± 4303
0.016 × 0.016 inches	7176 ± 4232	10,630 ± 5022	11,752 ± 4573 *^,+^	7184 ± 3395	8332 ± 4045
0.016 × 0.022 inches	5123 ± 3684	10,196 ± 5322+	9244 ± 3164 ^+^	11,244 ± 4366 *^,+^	8979 ± 3099
0.017 × 0.025 inches	6860 ± 4024	12,041 ± 5477 *^,+^	9724 ± 6290	8316 ± 3112	8865 ± 4348
Mean ± SD (*n* = 144 sites)	6210 ± 3363	9229 ± 3944	9256 ± 4288	7865 ± 3360	8152 ± 3968
Stainless steel (caliber)					
0.016 inches	7850 ± 4667	6757 ± 2258	7690 ± 4662	6452 ± 3751	7889 ± 4319
0.016 × 0.016 inches	6948 ± 5048	10,880 ± 3832+	7999 ± 3104	8214 ± 4115	7750 ± 3162
0.016 × 0.022 inches	8740 ± 6163	10,547 ± 5041	12,616 ± 4330 *^,+^	10,137 ± 4642	7272 ± 2982
0.017 × 0.025 inches	7542 ± 5288	11,641 ± 6222 *^,+^	12,821 ± 6005 *^,+^	10,586 ± 515 ^+^	7058 ± 3457
Mean ± SD (*n* = 96 sites)	7770 ± 5292	9956 ± 4338	10,281 ± 4525	8847 ± 4415	7492 ± 3480
Total mean ± SD (*n* = 240 sites)	6990 ± 4237	9592 ± 4614	9768 ± 4778	8356 ± 4123	7822 ± 3867
Mean ± SD for round arches (*n* = 96 sites)	6488 ± 3227	7317 ± 2525	8126 ± 4091	6724 ± 3259	7657 ± 4158
Mean ± SD for square–rectangle arches (*n* = 144 sites)	7064 ± 4740	10,989 ± 5153	10,692 ± 4578	9280 ± 4130	8043 ± 3516

*n* = 6 patients; SD, standard deviation; PU, relative perfusion units; min, minutes; h, hours; d, days; *n* = 24 sites of perfusion (vestibular papilla) (1 site on anterior teeth and 1 site on posterior teeth on the maxilla and mandible with a total of 4 sites per patient). Considering 6 patients, we obtained a total of 24 sites per arch, with a total of 240 sites for the 10 treatment arches. To establish the differences among variables in the study group, the Wilcoxon signed-rank test was used. * Statistically significant differences (*p* < 0.005) between the ITSRF with no treatment (7188 up) and that with treatment. ^+^ Statistically significant differences (*p* < 0.005) in ITSRF after 20 min compared to the different time intervals of all of the arches.

**Table 3:** Difference between integration (DBI) in patients with orthodontic treatment (Nitinol and stainless steel arches) at different time intervals.

Time Intervals	20 min	24 h	48 h	72 h	30 d
Arch groups (*n* = 24 sites by arch)			Mean ± SD (PU)		
Nitinol (caliber)					
0.012 inches	−1248 ± 1268	−947 ± 1259	−558 ± 1203	−826 ± 1163	−567 ± 1436
0.014 inches	−776 ± 729	−1062 ± 1827	−1276 ± 1554	−624 ± 1131	−758 ± 2747
0.016 inches	−502 ± 1248	−647 ± 910	−852 ± 1106	−643 ± 1173	−2178 ± 2921 *^,+^
0.016 × 0.016 inches	−1079 ± 1476	−703 ± 1830	−874 ± 1808	−272 ± 1488	−617 ± 2431
0.016 × 0.022 inches	−241 ± 1337	−1727 ± 2042	−701 ± 1490	−1046 ± 2214	−1190 ± 1329
0.017 × 0.025 inches	−147 ± 1467	−1234 ± 2949	−792 ± 1735	−761 ± 1392	−1007 ± 1434
Mean ± SD (*n* = 144 sites)	−665 ± 1254	−1053 ± 1803	−842 ± 1482	−696 ± 1427	−1052 ± 2049
Stainless steel (caliber)					
0.016 inches	−282 ± 1100	−215 ± 2058	−756 ± 1293	−283 ± 1094	−843 ± 1238
0.016 × 0.016 inches	−1401 ± 1591	−979 ± 1617	−1438 ± 1836	−1033 ± 2143	−355 ± 938
0.016 × 0.022 inches	−336 ± 1429	337 ± 1700	−1706 ± 2185	−535 ± 1806	−184 ± 896
0.017 × 0.025 inches	−978 ± 844	−999 ± 2506	−449 ± 2161	−1884 ± 1755	−994 ± 1417
Mean ± SD (*n* = 96 sites)	−750 ± 1241	−464 ± 1970	−1087 ± 1868	−968 ± 1699	−594 ± 1122
Total mean ± SD (*n* = 240 sites)	−536 ± 1243	−573 ± 1942	−724 ± 1804	−613 ± 1654	−881 ± 1276
Mean ± SD for round arches (*n* = 96 sites)	−702 ± 1086	−718 ± 1513	−861 ± 1289	−594 ± 1140	−1,087 ± 2085
Mean ± SD for square–rectangle arches (*n* =144 sites)	−697 ± 1357	−884 ± 2107	−994 ± 1869	−944 ± 1799	−725 ± 1407

*n* = 6 patients; SD, standard deviation; PU, relative perfusion units; min, minutes; h, hours; d, days; *n* = 24 sites of perfusion (vestibular papilla) (1 site on anterior teeth and 1 site on posterior teeth on the maxilla and mandible with a total of 4 sites per patient). Considering 6 patients, we obtained a total of 24 sites per arch, with a total of 240 sites for the 10 treatment arches. * Statistically significant differences (*p* < 0.005) in the DBI with no treatment (−524 up ± 3160 SD) and that with treatment. ^+^ Statistically significant differences (*p* < 0.005) in the DBI after 20 min compared to the time intervals of all of the arches.

**Table 4:** A time interval comparison (hours and days) of the variable difference between integration in patients with orthodontic treatment.

Difference Between Integration (DBI)	
Time interval (h) vs. time interval (days)	*p*
0.016-inch Nitinol arch	
24 h vs. 30 days	0.0171
48 h vs. 30 days	0.0493
72 h vs. 30 days	0.0168

Hrs, hours. To establish the differences between the time intervals for each arch, the Wilcoxon signed-rank test was used.

## Data Availability

The data supporting reported results can be found with the corresponding author.
